# Annexin A6 regulates catabolic events in articular chondrocytes via the modulation of NF-κB and Wnt/ß-catenin signaling

**DOI:** 10.1371/journal.pone.0197690

**Published:** 2018-05-17

**Authors:** Takeshi Minashima, Thorsten Kirsch

**Affiliations:** Musculoskeletal Research Center, Department of Orthopaedic Surgery, New York University School of Medicine, New York, New York, United States of America; National Cancer Center, JAPAN

## Abstract

Annexin A6 (AnxA6) is expressed in articular chondrocytes at levels higher than in other mesenchymal cell types. However, the role of AnxA6 in articular chondrocytes is not known. Here we show that complete lack of AnxA6 functions resulted in increased ß-catenin activation in Wnt3a-treated murine articular chondrocytes, whereas AnxA6 expressing articular chondrocytes showed decreased ß-catenin activation. High expression of AnxA6 in human articular chondrocytes showed the highest inhibition of Wnt/ß-catenin signaling. Inhibition of Wnt/ß-catenin signaling activity by AnxA6 together with cytosolic Ca^2+^ was achieved by interfering with the plasma membrane association of the Wnt signaling complex. AnxA6 also affected the cross-talk between Wnt/ß-catenin signaling and NF-κB signaling by decreasing ß-catenin activity and increasing NF-κB activity in Wnt3a-, interleukin-1beta (IL-1ß)-, and combined Wnt3a/IL-1ß-treated cells. Wnt3a treatment increased the mRNA levels of catabolic markers (cyclooxygenase-2, interleukin-6, inducible nitric oxide synthase) to a much lesser degree than IL-1ß treatment in human articular chondrocytes, and decreased the mRNA levels of matrix metalloproteinase-13 (MMP-13) and articular cartilage markers (aggrecan, type II collagen). Furthermore, Wnt3a decreased the mRNA levels of catabolic markers and MMP-13 in IL-1ß-treated human articular chondrocytes. High expression of AnxA6 resulted in decreased mRNA levels of catabolic markers, and increased MMP-13 and articular cartilage marker mRNA levels in Wnt3a-treated human articular chondrocytes, whereas leading to increased mRNA levels of catabolic markers and MMP-13 in human articular chondrocytes treated with IL-1ß, or combined Wnt3a and IL-1ß. Our findings define a novel role for AnxA6 in articular chondrocytes via its modulation of Wnt/ß-catenin and NF-κB signaling activities and the cross-talk between these two signaling pathways.

## Introduction

Currently, very little is known about the role of annexins in articular cartilage despite the rapid progress in the understanding of the role of annexins in various other tissues and diseases, including Alzheimer’s disease, autoimmunity, cancer, diabetes, and cardiovascular diseases [1–4]. Studies have shown that annexins play major roles in various tissues and diseases via acting as intracellular modulators of various signaling pathways, including signaling pathways which play major roles in cartilage homeostasis, maintenance and pathology [1–4]. A previous study has shown that various annexins, including AnxA6, are expressed in articular chondrocytes at higher levels than in other mesenchymal cell types [5]. However, the role of AnxA6 in articular chondrocytes is not well understood.

In recent years, the NF-κB and the canonical Wnt signaling pathways have been implicated to play an important role in cartilage homeostasis, maintenance and the pathophysiology of OA [6–10]. Pro-inflammatory cytokines such as interleukin-1 (IL-1) are potent inducers of cartilage degradation via the activation of NF-κB signaling in articular chondrocytes, which result in the expression of MMPs, such as MMP-13 [11–13]. Previous studies have shown that low levels of Wnt/ß-catenin signaling are required for maintenance of normal articular cartilage function and that deregulation of this pathway contributes to the development and progression of cartilage degeneration and OA pathology [6, 8, 9, 14, 15, 16]. Therefore, signaling pathways, such as NF-κB and Wnt/ß-catenin signaling pathways, have to be tightly regulated, and de-regulation of these pathways can have severe consequences.

The different signaling pathways involved in cartilage homeostasis and OA pathogenesis, including NF-κB and Wnt/ß-catenin signaling do not act in isolation but these signaling pathways interact and affect each other. In addition, these signaling pathways may also interact with each other in tissue or cell type-specific manners. Finally, it is not just the activation of a signaling pathway that contributes to disease pathology but also the degree and duration of activation. For example, as discussed above, low degree of canonical Wnt/ß-catenin signaling activation was suggested to be required for long-term maintenance and function of articular cartilage, whereas prolonged or chronic activation of Wnt/ß-catenin signaling has been shown to cause cartilage degradation and joint disease [8, 9, 14, 15, 16]. Furthermore, a recent study demonstrated that Wnt/ ß -catenin signaling interacts with NF-κB signaling in human articular chondrocytes [17]. Specifically, this study showed that Wnt/ß-catenin signaling in human articular chondrocytes inhibits MMP expression via counteracting pro-catabolic NF-κB signaling, whereas Wnt/ß-catenin signaling stimulated MMP expression in murine and bovine articular chondrocytes [17].

We and others have shown that annexins act as intracellular modulators of various signaling pathways [17–19]. For example, we have shown that AnxA6 stimulates NF-κB signaling via interaction with the p65 subunit of the active NF-κB complex leading to increased nuclear translocation and retention of the active p50/p65 NF-κB complex [20]. Since AnxA6 in the presence of Ca^2+^ can bind to the inner leaflet of the plasma membrane [21], we hypothesized that AnxA6 in articular chondrocytes may inhibit the activation of the Wnt/ß-catenin signaling complex via interfering with the association of this complex with the plasma membrane, which is required for the activation of Wnt/ß-catenin signaling [22, 23, 24]. Previous studies have shown that the DEP domain located in C-terminal half of Dvl1 is critical for plasma membrane recruitment of Dvl1 during Wnt/ß-catenin signaling [22, 24, 25]. Furthermore, we asked the question whether AnxA6 affects catabolic events in human articular chondrocytes via directly stimulating NF-κB signaling and indirectly via the inhibition of Wnt/ß-catenin signaling and counteracting role of Wnt/ß-catenin signaling on NF-κB signaling.

## Materials and methods

### Mice

The AnxA6-/- mice were provided to us by Dr. S. E. Moss, University College of London, UK. These mice have a C57BL/6 genetic background, and mice heterozygous for the mutation in AnxA6 were used for breeding [26]. All protocols involving mice were approved by the Institutional Animal Care and Use Committee and NYU School of Medicine. Mice were euthanized by carbon dioxide inhalation (non-charged method). This slow hypoxia method minimizes animal distress and is approved by the Panel of Euthanasia of the American Veterinary Medical Association. Animals were observed to ensure that they become prostrate and allowed to remain in the chamber for at least 1 minute after apparent clinical death. Cervical dislocation was performed on all animals to ensure death. All euthanasia procedures were performed solely within the animal facility.

### Cell cultures

Murine articular chondrocytes were isolated from the articular cartilage cap of the femoral head, femoral condyles and tibial plateau of 21–day-old AnxA6-/- mice and WT littermates as described previously [27]. Cells were plated at a density of 1 x 10^6^ / well into 6-well tissue culture plates and grown in monolayer cultures in Dulbecco’s modified Eagle’s medium (DMEM; Life Technologies, Gaithersburg, MD) containing 10% fetal calf serum (FCS; HyClone, Logan, Utah), 2mM L-glutamine (Invitrogen, Carlsbad, CA), and 50U/ml of penicillin and streptomycin (Invitrogen) (complete medium). Human articular chondrocytes were isolated from articular cartilage samples obtained from patients (donor age range 48–67) undergoing total knee replacement surgery at NYU Langone Orthopedic Hospital. Knee cartilage was harvested from regions with no macroscopically evident degeneration. The collection of tissue from patients undergoing knee replacement surgery was ethically approved by the Institutional Regulatory Board at NYU School of Medicine. Tissue samples were obtained after informed written consent was obtained. Human chondrocytes were isolated from these cartilage samples as described by us [28]. Cells were plated at density of 1 x 10^6^ cells/well into 6-well tissue culture plate and cultured in complete medium as described above. Semiconfluent human or mouse chondrocytes were transfected with empty pcDNA expression vector or pcDNA expression vector containing AnxA6 cDNA (pcDNA-AnxA6) using X-tremeGene HP DNA transfection reagent per the manufacturer’s protocol (Roche, Branchburg, NJ). Briefly, 200 μl Opti-MEM reduced serum medium containing 2 μg plasmid DNA together with 6 μl X-tremeGENE HP DNA transfection reagent were incubated for 30 min at room temperature. After incubation, the transfection complex was added to the cells in a drop-wise manner. Cells were incubated for 24 h at 37°C followed by serum starvation. Twenty four hours after serum starvation cells were pretreated for 2 h either with vehicle (dimethyl sulfoxide; DMSO) or 1,2-bis(2-aminophenoxy)ethane-N,N,N’,N’-tetraacetic acid tetra(acetoxymethyl) ester (BAPTA)-2AM, a cell permeant Ca^2+^ chelator (10μM; Thermo Fisher Scientific) followed by treatment with Wnt3a (200ng/ml; R&D Systems, Minneapolis, MN) and/or IL-1ß (10ng/ml) for various time points. The concentrations used for Wnt3a and IL-1ß are the same concentrations, which have been used in previous studies, including the study by Ma et al. (17). We used the pcDNA vector, which contains a *c-myc* tag. We obtained a transfection rate between 50% to 70% as determined by immunostaining of transfected cells with FITC-labeled antibodies specific for c-Myc (ab39688, Abcam, Cambridge, MA) and counterstaining of cell nuclei with DAPI (data not shown).

### Luciferase reporter assays

For luciferase assays to determine ß-catenin activity, cells were co-transfected with a firefly TCF/LEF-specific luciferase reporter vector (TOPFlash Reporter; EMD Millipore, Billerica, MA) or FOPFlash reporter, which contains a mutated TCF/LEF binding site and therefore serves as a negative control and a constitutively expressed Renilla luciferase reporter, which served as an internal control for normalizing transfection efficiencies and monitoring cell viability. Luciferase activities from both reporters were measured using a dual luciferase assay kit (Promega Corp., Madison, WI). For luciferase assays to determine NF-κB activity, cells were co-transfected with a firefly NF-κB-specific luciferase reporter vector (pNFκB-Met-Luc2-Reporter; Clontech Laboratories Inc., Mountain View, CA). Luciferase activity in the medium from the secreted Metridia luciferase reporter gene was monitored using the Ready-To-Glow Secreted Luciferase Reporter Assay (Clontech). Transfection efficiency was monitored by co-transfection with pSEAP vector (Clontech), which provides constitutive expression of secreted form of human placental alkaline phosphatase (SEAP). Secreted SEAP activity was monitored with the chemiluminescent substrate CSPD (Clontech). Luciferase activities were measured using the Tristar LB 941 luminometer (Berthold Technologies, Oak Ridge, TN) and was measured 48 h after transfection with the luciferase reporters. All experiments were performed in triplicate and repeated three to five times.

### RT-PCR and real-time PCR analysis

Total RNA was isolated from chondrocyte cultures using the RNeasy minikit (Qiagen, Valencia, CA). mRNA levels of aggrecan, cyclooxygenase (Cox)-2, IL-6, inducible nitric oxide synthase (iNOS), MMP-13, and type II collagen were quantified by real-time PCR as described previously [29]. Briefly, 1 μg of total RNA was reverse transcribed in a total volume of 30 μl by using an Omniscript RT kit (Qiagen). A 1:100 dilution of the cDNA resulting from the reverse transcription of 1 μg of total RNA was used as the template to quantify the relative content of mRNA by real-time PCR (StepOnePlus^TM^ System; Applied Biosystems, Foster City, CA) with the appropriate primers and SYBR Green. PCRs were performed with a SYBR Green PCR Master Mix kit (Applied Biosystems), with 95°C for 10 min followed by 40 cycles at 95°C for 15 s and 60°C for 1 min, and 1 cycle at 95°C for 15 s and 60°C for 1 min. The 18S RNA was amplified at the same time and used as an internal control. The cycle threshold values for 18S RNA and the samples were measured and calculated by computer software. Relative transcript levels were calculated as x = 2^–ΔΔCt^, in which ΔΔCt = ΔE– ΔC, ΔE = Ct_exp_−Ct_18S,_ and ΔC = Ct_ctl_−Ct_18S._

### Total lysates and subcellular fractionation

Total lysates were obtained from human articular chondrocyte cultures 48 h after transfection with empty pcDNA expression vector of pcDNA expression vector containing full length AnxA6 cDNA or from late stage human OA articular cartilage using T-PER^TM^ Tissue Protein Extraction Reagent from Pierce Biology Products. To extract the nuclear and plasma membrane proteins from the cell cultures we used the NE-PER^TM^ Nuclear and Cytoplasmic Extraction Reagent or the Mem-PER^TM^ Plus Membrane Protein Extraction Kit from Pierce Biology Products and followed the manufacturer’s instructions. Thirty μg of total cytoplasmic, nuclear or plasma membrane protein fractions were analyzed by SDS PAGE and immunoblotting with antibodies specific for AnxA6 (Abcam, ab7671), ß-catenin (Abcam, ab6302), Dishevelled 1 (Dvl1, Abcam, ab174679) as described previously [20]. For normalization of the protein expression levels, the membranes were immunostained with antibodies specific for beta-actin (total tissue cell lysate, Abcam, ab20272), alpha 1 sodium potassium ATPase (ATP1A1, plasma membrane fraction, Abcam, ab7671) and lamin B (nuclear fraction, Abcam, ab194109).

### Statistical analysis

The data are expressed as mean ± standard deviation (SD). SPSS software 19.0 and GraphPad Prism 6.0 were used to analyze the data. Analysis of the variance (ANOVA) was used to assess differences among 3 or more groups, while comparison of two groups was made using unpaired t tests. If there were significant differences in ANOVA, pairwise tests were conducted to assess specific differences using Tukey’s multiple comparison procedure. A p value below 0.05 was considered statistically significant.

## Results

### The effect of AnxA6 function on Wnt/ß-catenin signaling in articular chondrocytes

**Fig 1:**
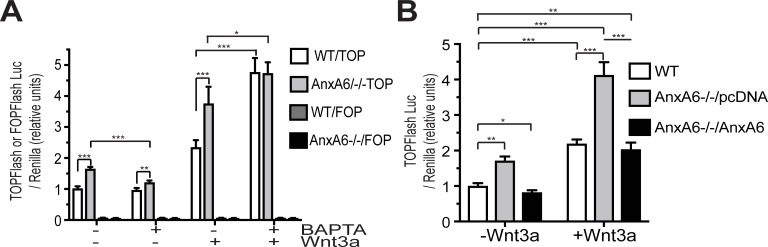
ß-catenin activity in mouse AnxA6-/- and WT articular chondrocytes. **A:** ß-catenin activity was determined by luciferase activity from the TOPFlash (TOP) or FOPFlash (FOP) luciferase reporter in vehicle-treated, Wnt3a-treated, and BAPTA-2AM/Wnt3a-treated WT and AnxA6-/- mouse articular chondrocytes. **B:** ß-catenin activity was determined by luciferase activity from the TOPFlash (TOP) or FOPflash (FOP) luciferase reporter in vehicle-treated, and Wnt3a-treated WT or AnxA6-/- mouse articular chondrocytes that were transfected with empty pcDNA expression vector (pcDNA) or pcDNA expression vector containing full-length AnxA6 cDNA (AnxA6). Mouse articular chondrocytes after transfection in **A** and **B** were serum starved followed by treatment with Wnt3a or the intracellular Ca^2+^ chelator, BAPTA-2AM followed by Wnt3a. Cell extracts were then analyzed for luciferase activity 48 h post-transfection. Transfection efficiency was monitored by co-transfection with Renilla luciferase vector, which provides constitutive expression of the Renilla luciferase reporter. Values were normalized to Renilla luciferase activity. Data are expressed relative to the normalized luciferase activity levels from the TOPFlash reporter of vehicle-treated WT cells, which was set as 1. Data (*n* = 4) are expressed as mean ± SD. (*p < 0.05; **p < 0.01; ***p < 0.001).

To determine how AnxA6 affects Wnt/ß-catenin signaling, we used articular chondrocytes isolated from AnxA6-/- mice. Wnt3a treatment increased Wnt/ß-catenin signaling as indicated by increased luciferase activity from the TOPFlash reporter in AnxA6-/- articular chondrocytes and WT cells. Complete absence of AnxA6 resulted in increased Wnt/ß-catenin signaling as indicated by the increased luciferase activity from the TOPFlash reporter in vehicle-treated and Wnt3a-treated articular chondrocytes isolated from AnxA6-/- mice compared to vehicle-treated and Wnt3a-treated chondrocytes isolated from WT littermates. Transfection with FOPFlash reporter that encodes mutated LEF/TCF binding sites elicited no response (Fig 1A). These findings demonstrate that AnxA6 inhibits Wnt/ß-catenin signaling.

Since AnxA6 requires Ca^2+^ to associate with the plasma membrane [30], we determined the role of intracellular Ca^2+^ together with AnxA6 on the inhibition of Wnt/ß-catenin signaling. We first transfected WT and AnxA6-/- articular chondrocytes with the TOPFlash luciferase reporter. Twenty-four hours after transfection these cells were treated with the cytoplasmic Ca^2+^ chelator BAPTA-2AM followed by treatment with Wnt3a. Treatment of WT chondrocytes with BAPTA-2AM showed similar luciferase activity from the TOPFlash reporter as vehicle-treated cells, while BAPTA-2AM-treated AnxA6-/- chondrocytes showed lower luciferase activity compared to vehicle-treated AnxA6-/- cells. In the presence of BAPTA-2AM the luciferase activity from the TOPFlash reporter was increased in Wnt3a-treated WT and AnxA6-/- cells compared to Wnt3a-treated cells in the absence of BAPTA-2AM. This increase in AnxA6-/- cells, however, was less pronounced than the increase in WT cells. BAPTA-2AM resulted in a similar luciferase activity in Wnt3a-treated WT cells as in Wnt3a-treated AnxA6-/- chondrocytes. Transfection with FOPFlash reporter elicited no response (Fig 1A). These findings provide evidence that AnxA6 together with cytoplasmic Ca^2+^ regulate Wnt/ß-catenin signaling.

Rescue experiments revealed that transfection of AnxA6-/- chondrocytes with an expression vector containing full-length AnxA6 cDNA showed reduced luciferase activity from the TOPFlash reporter in the absence or presence of Wnt3a compared to AnxA6-/- chondrocytes transfected with empty expression vector. TOPFlash luciferase activity in AnxA6-/- chondrocytes transfected with expression vector containing full-length AnxA6 was similar to the levels in WT chondrocytes (Fig 1B).

### AnxA6 interferes with plasma membrane association of the Wnt signaling complex

**Fig 2:**
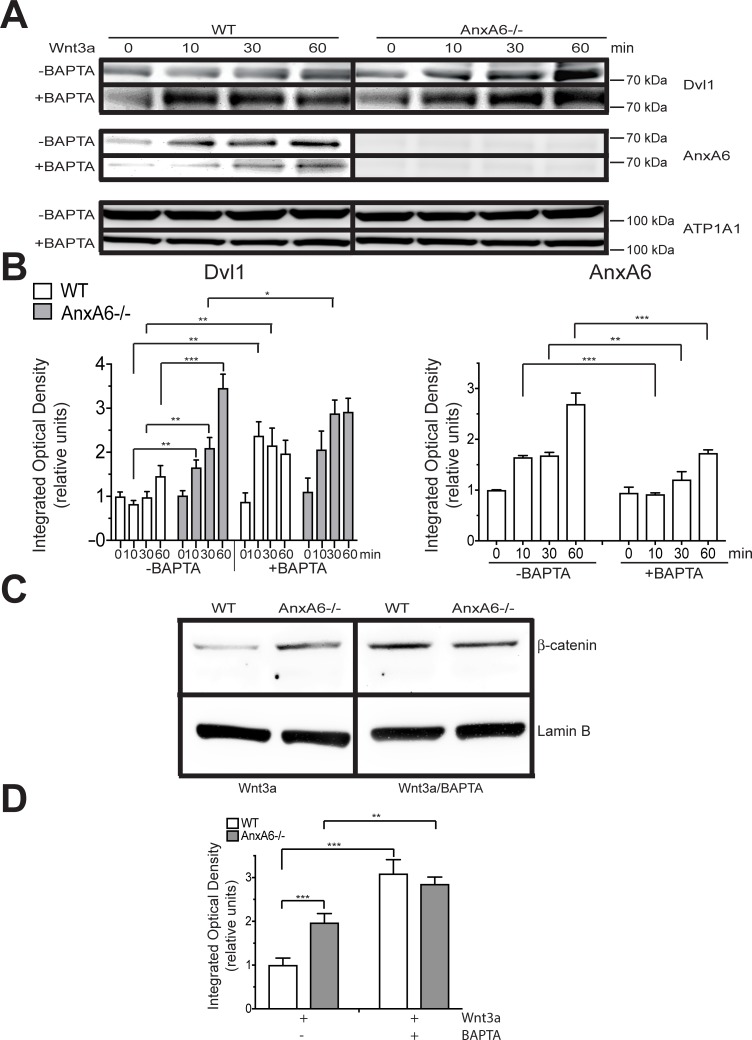
AnxA6 interferes with plasma membrane association of Dvl1 in a Ca^2+^-dependent manner. **A:** Immunoblot analysis of the plasma membrane fraction isolated from WT and AnxA6-/- mouse articular chondrocytes treated with Wnt3a for the time periods indicated, or treated with the intracellular Ca^2+^ chelator, BAPTA-2AM followed by Wnt3a treatment, and analyzed with antibodies specific for Dvl1and AnxA6. Blots were also analyzed with antibodies specific for ATP1A1 (a plasma membrane protein) to control for equal loading. **B:** The optical densities of the Dvl1 and AnxA6 bands were quantitated by densitometry and normalized to the optical densities of the ATP1A1 bands. Results are expressed relative to the normalized optical densities of the Dvl1 or AnxA6 bands of vehicle-treated WT cells, which were set as 1. **C:** Immunoblot analysis of the nuclear fraction isolated from WT and AnxA6-/- mouse articular chondrocytes treated with Wnt3a for 30 min, or treated with the intracellular Ca^2+^ chelator, BAPTA-2AM followed by Wnt3a treatment, and analyzed with antibodies specific for ß-catenin. Blots were also analyzed with antibodies specific for lamin B (a nuclear protein) to control for equal loading. **D:** The optical densities of the ß-catenin bands were quantitated by densitometry and normalized to the optical densities of the lamin B bands. Results are expressed relative to the normalized optical densities of the ß-catenin bands of Wnt3a-treated WT cells, which were set as 1. The blots in **A** and **C** are representative of 3 separate experiments with similar results. The optical densities were analyzed on three different immunoblots, and values are mean ± SD. (*p < 0.05; **p < 0.01; ***p < 0.001).

Next, we determined the mechanism of how AnxA6 inhibits Wnt/ß-catenin signaling. The activation of Wnt signaling requires the formation of a signaling complex mediated by Dvl and the plasma membrane association of this complex [22–25]. Specifically, the DEP domain located in C-terminal half of Dvl1 is critical for plasma membrane recruitment of Dvl1 during Wnt/ß-catenin signaling [22–25]. To determine whether plasma membrane-associated AnxA6 interferes with the plasma membrane association of the Wnt signaling complex, we treated WT and AnxA6-/- chondrocytes with canonical Wnt3a for various time periods (0, 10, 30, and 60 min) and analyzed the amounts of AnxA6 and Dvl1 in the plasma membrane fraction using immunoblot analysis. Wnt3a-treated WT articular chondrocytes contained higher amounts of plasma membrane-associated AnxA6 than untreated WT cells, whereas no immunostaining for AnxA6 was detectable in the plasma membrane fraction of AnxA6-/- chondrocytes. Markedly more Dvl1 was detected in the plasma membrane fraction of AnxA6-/- chondrocytes treated for 10min, 30min or 60min with Wnt3a than in this fraction of Wnt3a-treated WT cells (Fig 2A and 2B). These findings reveal that AnxA6 inhibits canonical Wnt/ß-catenin signaling via interfering with the plasma membrane association of the Wnt signaling complex.

To determine the role of cytoplasmic Ca^2+^ in the plasma membrane association of AnxA6 and ultimately its ability to compete with the plasma membrane association of the Wnt signaling complex, we treated WT and AnxA6-/- articular chondrocytes with BAPTA-2AM for 2 h followed by treatment with Wnt3a for various time points (0, 10, 30, and 60 min) and analyzed the amounts of AnxA6 and Dvl1 in the plasma membrane fraction by immunoblot analysis. Wnt3a treatment of BAPTA-2AM-treated WT chondrocytes resulted in decreased amounts of AnxA6 in the plasma membrane fraction compared to WT cells not treated with BAPTA-2AM. No immunostaining for AnxA6 was obtained in the plasma membrane fraction of AnxA6-/- chondrocytes treated with BAPTA-2AM and Wnt3a. Furthermore, BAPTA-2AM resulted in increased amounts of Dvl1 in the plasma membrane fraction of WT chondrocytes to levels similar to the amount of Dvl1 in the plasma membrane fraction of BAPTA-2AM / Wnt3a-treated AnxA6-/- chondrocytes (Fig 2A and 2B).

Activation of Wnt/ß-catenin signaling leads to the inactivation of ß-catenin degradation and the subsequent accumulation of ß-catenin in the nucleus [31, 32]. Wnt3a treatment for 30 min resulted in increased amounts of nuclear ß-catenin in AnxA6-/- chondrocytes compared to WT cells. BAPTA-2AM treatment, however, resulted in similar amounts of nuclear ß-catenin in Wnt3a-treated WT and AnxA6-/- chondrocytes (Fig 2C and 2D). These findings demonstrate that Ca^2+^ is required for the plasma membrane association of AnxA6, the interference of AnxA6 with the plasma membrane association of the Wnt signaling complex, and ultimately the inhibition of Wnt/ß-catenin signaling by AnxA6.

### The effect of AnxA6 on the cross-talk between NF-κB and Wnt/ß-catenin signaling in human articular chondrocytes

**Fig 3:**
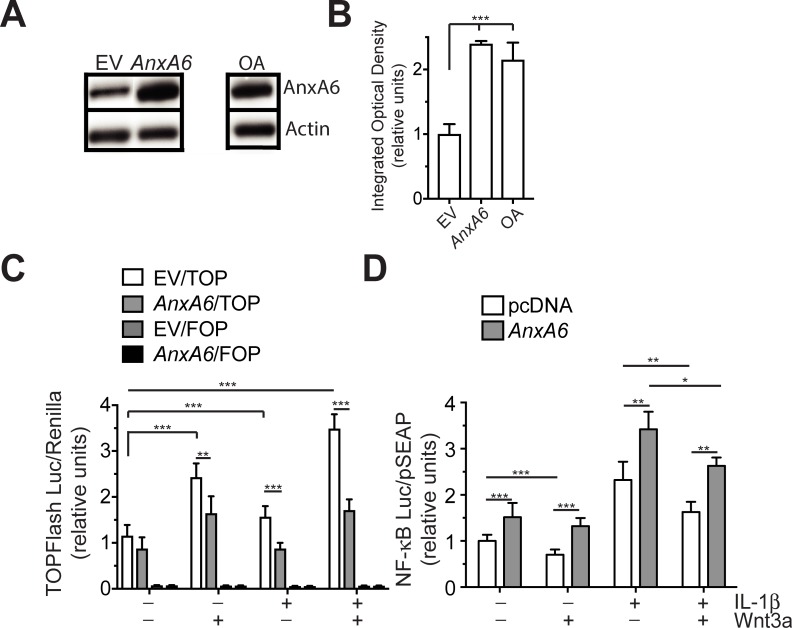
AnxA6 modulates the crosstalk between NF-κB and ß-catenin signaling activities. **A:** Immunoblot analysis with antibodies specific for AnxA6 of a lysate from human articular chondrocytes transfected with empty pcDNA expression vector (EV) or pcDNA expression vector containing full-length *AnxA6* cDNA (*AnxA6*), or a lysate from late stage OA human articular cartilage tissue. Late stage OA cartilage was defined as deep fibrillated cartilage with loss of cartilage of the superficial and middle zones. Blots were also analyzed with antibodies specific for beta-actin to control for equal loading. **B:** The optical densities of the AnxA6 bands were quantitated by densitometry and normalized to the optical densities of the actin bands. Results are expressed relative to the normalized optical densities of the AnxA6 bands of human articular chondrocytes transfected with empty expression vector (EV), which were set as 1. The blot in **A** is representative of 3 separate experiments with similar results. The optical densities were analyzed on three different immunoblots, and values are mean ± SD. **C:** ß-catenin activity as determined by luciferase activity from the TOPFlash (TOP) or FOPFlash (FOP) luciferase reporter in vehicle-treated, Wnt3a-treated, IL-1ß-treated, Wnt3a/IL-1ß-treated human articular chondrocytes overexpressing AnxA6. After serum starvation, TOPFlash or FOPFlash reporter- and empty pcDNA (pcDNA)-transfected, or TOPFlash or FOPFlash reporter- and pcDNA containing full-length *AnxA6* (*AnxA6*)-transfected human articular chondrocytes were vehicle-treated, or treated with Wnt3a, IL-1ß, or Wnt3a and IL-1ß for 24h. Cell extracts were then analyzed for luciferase activity. Transfection efficiency was monitored by co-transfection with Renilla luciferase vector, which provides constitutive expression of the Renilla luciferase reporter. **D:** NF-κB activity as determined by luciferase activity from the pNFκB-Met-Luc2 reporter in vehicle-treated, Wnt3a-treated, IL-1ß-treated, Wnt3a/IL-1ß-treated human articular chondrocytes overexpressing AnxA6. After serum starvation, pNFκB-Met-Luc2 reporter- and empty pcDNA (pcDNA)-transfected, or pNFκB-Met-Luc2 reporter- and pcDNA containing full-length *AnxA6* (*AnxA6)*-transfected human articular chondrocytes were vehicle-treated, or treated with Wnt3a, IL-1ß, or Wnt3a and IL-1ß for 24h. Samples were then analyzed for luciferase activity. Transfection efficiency was monitored by co-transfection with pSEAP vector, which provides constitutive expression of human placental alkaline phosphatase (SEAP). Secreted SEAP activity was monitored with the chemiluminescence substrate CSPD. Values in **C** and **D** were normalized to Renilla or pSEAP luciferase activity. Data are expressed relative to the normalized luciferase activity levels of vehicle-treated WT cells transfected with empty expression vector, which was set as 1. Data in **C** and **D** (*n* = 4) are expressed as mean ± SD. (*p < 0.05; **p < 0.01; ***p < 0.001).

A previous study has shown that IL-1ß activates NF-κB signaling and Wnt/ß-catenin signaling [16]. In addition, the same study showed that Wnt/ß-catenin signaling inhibited NF-κB signaling in human articular chondrocytes [17]. We determined whether AnxA6 in human articular chondrocytes affects the crosstalk between NF-κB and Wnt/ß-catenin signaling. Overexpression of AnxA6 using the pcDNA expression vector resulted in similar amounts of AnxA6 protein in human articular chondrocytes as in extracts from late-stage OA cartilage. The amount of AnxA6 protein in extracts from human articular chondrocytes transfected with empty pcDNA expression vector was markedly less than the amount of AnxA6 protein in extracts from human articular chondrocytes transfected with pcDNA expression vector containing full length AnxA6 cDNA or late-stage OA cartilage (Fig 3A and 3B).

Vehicle-treated chondrocytes transfected with expression vector containing *AnxA6* showed a reduced but not statistically significant reduction in luciferase activity from the TOPFlash reporter compared to vehicle-treated cells transfected with empty expression vector. Wnt/ß-catenin signaling, however, was significantly (p < 0.01) reduced in Wnt3a-treated human articular chondrocytes transfected with *AnxA6* containing expression vector compared to Wnt3a-treated cells transfected with empty expression vector. IL-1ß stimulated Wnt/ß-catenin signaling activity in human articular chondrocytes transfected with empty expression vector by ~1.5-fold compared to vehicle-treated human articular chondrocytes transfected with empty expression vector, whereas IL-1ß did not stimulate Wnt/ß-catenin signaling in human articular chondrocytes transfected with *AnxA6* containing expression vector. The combined Wnt3a/IL-1ß treatment resulted in the highest activation of Wnt/ß-catenin signaling activity in human articular chondrocytes transfected with empty expression vector (~3.5-fold increase compared to vehicle-treated cells), whereas the combined Wnt3a/IL-1ß treatment did not further increase ß-catenin signaling activity in human articular chondrocytes transfected with *AnxA6* containing expression vector compared to cells transfected with *AnxA6* containing expression vector and treated with Wnt3a only. Very little luciferase activity and little or no changes with the various treatments were measured in cells transfected with FOPFlash, a negative control reporter (Fig 3C).

Contrary, high expression of AnxA6 resulted in increased NF-κB activity compared to empty vector-transfected cells in the absence or presence of IL-1ß. These findings confirm our previous findings showing that AnxA6 stimulates NF-κB activity via binding to the p65 subunit of the active NF-κB complex [20]. Wnt3a treatment significantly (p < 0.003) reduced NF-κB activity in human articular chondrocytes transfected with empty expression vector, but not in cells transfected with AnxA6 containing expression vector. Furthermore, luciferase activity from the NF-κB luciferase reporter was reduced by the combined treatment with Wnt3a and IL-1ß compared to IL-1ß treatment alone in cells transfected with empty expression vector or AnxA6 containing expression vector; the activity from the NF-κB luciferase reporter, however, still was significantly (p < 0.01) higher in IL-1ß/Wnt3a-treated chondrocytes transfected with AnxA6 containing expression vector compared to cells transfected with empty expression vector (Fig 3D). These findings demonstrate that Wnt/ß-catenin signaling inhibits NF-B activity and that increased AnxA6 expression in human articular chondrocytes stimulates NF-κB signaling most likely directly via binding to p65 as shown by us previously [20], and indirectly via the inhibition of Wnt/ß-catenin signaling.

### The effect of high AnxA6 expression on the mRNA levels of articular cartilage and catabolic markers in human articular chondrocytes treated with Wnt3a, IL-1ß, or Wnt3a and IL-1ß

**Fig 4:**
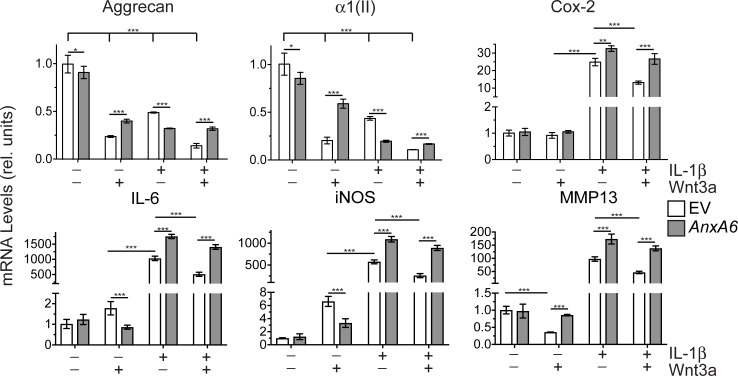
mRNA levels for articular cartilage markers (aggrecan, type II collagen (α1(II)) and catabolic markers (Cox-2, IL-6, iNOS, MMP-13) in vehicle-treated, Wnt3a-treated, IL-1ß-treated, or combined Wnt3a- and IL-1ß-treated human articular chondrocytes overexpressing AnxA6. Human articular chondrocytes were transfected with empty expression vector (EV) or expression vector containing full-length AnxA6 (*AnxA6*). Twelve hours after transfection cells were serum-starved for 24 h followed by treatment with IL-1ß, Wnt3a, IL-1ß, or combined IL-1ß and Wnt3a for 24h. Levels of mRNA were determined by real-time PCR using SYBR Green and normalized to the level of 18S RNA. The mRNA levels are expressed relative to the level of vehicle-treated cells transfected with empty expression vector, which was set as 1. Data were obtained from triplicate PCRs using RNA from 3 different cultures (*n* = 3). Values are the mean ± SD. (**p < 0.01; ***p < 0.001).

In the final set of experiments we determined how the inhibition of Wnt/ß-catenin signaling by AnxA6 affects catabolic events in human articular chondrocytes treated with Wnt3a, IL-1ß or a combination of Wnt3a and IL-1ß. Wnt3a treatment, IL-1ß treatment and the combined Wnt3a/IL-1ß treatment decreased the mRNA levels of aggrecan and type II collagen compared to the levels in vehicle-treated cells with the combined Wnt3a/IL-1ß treatment being the most effective treatment in reducing aggrecan and type II collagen mRNA levels. Wnt3a treatment of human articular chondrocytes increased the mRNA levels of the catabolic markers IL-6 and iNOS compared to vehicle-treated cells. Cox-2 mRNA levels were not affected by Wnt3a treatment, whereas MMP-13 mRNA levels were decreased. IL-1ß treatment resulted in a more pronounced increase in the mRNA levels of IL-6 and iNOS compared to Wnt3a-treated cells. In addition, IL-1ß treatment resulted in a marked increase in the mRNA levels of Cox-2 and MMP-13 compared to vehicle-treated cells. The combined treatment with Wnt3a and IL-1ß resulted in a decrease of the mRNA levels of Cox-2, IL-6, iNOS and MMP-13 compared to the levels in cells treated with IL-1ß only (Fig 4). These findings show that Wnt/ß-catenin signaling stimulated the mRNA levels of catabolic markers to a much lesser degree than IL-1ß. Consistent with a previous study [17], our findings show that Wnt/ß-catenin signaling inhibited the mRNA levels of MMP-13. In the presence of IL-1ß, however, Wnt/ß-catenin signaling reduced the expression of catabolic markers and MMP-13 in human articular chondrocytes.

Transfection of human articular chondrocytes with an expression vector containing full length AnxA6 resulted in a ~3 to 4-fold increased AnxA6 protein expression levels mimicking the high AnxA6 protein expression levels in late stage OA cartilage (see Fig 3A and 3B). High expression of AnxA6, which resulted in decreased Wnt/ß-catenin signaling (see Fig 3C), led to increased mRNA levels of aggrecan, type II collagen, and MMP-13 and decreased mRNA levels of IL-6 and iNOS in Wnt3a-treated human articular chondrocytes compared to the levels in Wnt3a-treated chondrocytes transfected with empty expression vector. Treatment of human articular chondrocytes transfected with expression vector containing AnxA6 with IL-1ß resulted in increased mRNA levels of catabolic markers (Cox2, IL-6, iNOS), and MMP-13 compared to IL-1ß-treated cells transfected with empty expression vector, while further decreasing the mRNA levels of articular cartilage markers, aggrecan and type II collagen. Furthermore, high expression of AnxA6 resulted in increased mRNA levels of articular cartilage markers (aggrecan and type II collagen) and catabolic markers (Cox2, IL-6, iNOS, MMP-13) in human articular chondrocytes, which received a combined IL-1ß and Wnt3a treatment compared to cells transfected with empty expression vector and treated with IL-1ß and Wnt3a (Fig 4). These findings show that high expression of AnxA6 stimulates the expression of catabolic markers in human articular chondrocytes treated with IL-1ß, or Wnt3a together with IL-1ß, while inhibiting the effects of Wnt3a treatment on catabolic and articular cartilage markers.

## Discussion

In this study we present evidence that AnxA6 is a novel regulator of the canonical Wnt signaling pathway in articular chondrocytes. Specifically, we show that plasma membrane-associated AnxA6 competes with the plasma membrane association of the Wnt signaling complex that contains Dvl, axin and adenomatosis polyposis coli (APC). Formation of this signaling complex and its association with the plasma membrane leads to the inactivation of GSK3ß, the inactivation of ß-catenin degradation and the subsequent accumulation of ß-catenin in the nucleus [22, 23, 31, 32].

Annexins, including AnxA6, are cytoplasmic proteins present in a variety of cell types, including chondrocytes [21, 33]. In the presence of Ca^2+^ these annexins interact with phospholipids and associate with cellular membranes, including the plasma membrane [21]. A previous study has shown that different annexins require different intracellular Ca^2+^ concentrations to associate with various cellular membranes [34]. Our study showing that the amount of Dvl1 in the plasma membrane fraction was increased in Wnt3a-treated human articular chondrocytes when intracellular Ca^2+^ was chelated by BAPTA-2AM, while AnxA6 was decreased demonstrates that cytoplasmic Ca^2+^ is required for AnxA6 to associate with the plasma membrane of chondrocytes and ultimately to inhibit Wnt/ß-catenin signaling. These findings suggest that cytoplasmic Ca^2+^ plays a major regulatory role in AnxA6-mediated inhibition of Wnt/ß-catenin signaling in articular chondrocytes.

Our study shows that the amount of plasma membrane-associated AnxA6 increased in Wnt3a-treated WT chondrocytes compared to vehicle-treated WT cells. Recently, it was shown that Wnt3a stimulates the canonical and non-canonical pathways in mesenchymal stem cells and chondrocytes [35, 36]. The non-canonical Wnt signaling pathway activates the Ca^2+^/calmodulin-dependent protein kinase II pathway, which results in an increase in intracellular Ca^2+^ concentration [37]. In addition, a previous study has shown that IL-1 treatment leads to a transient increase in the intracellular Ca^2+^ concentration in articular chondrocytes [38]. These studies together with our findings suggest that Wnt3a and IL-1 treatment via transiently increasing intracellular Ca^2+^ concentration enable increased amounts of AnxA6 to associate with the plasma membrane resulting in the inhibition of Wnt/ß-catenin signaling.

A recent study showed that several annexins, including AnxA6 are expressed at higher levels in articular chondrocytes than in other mesenchymal cell types [5]. Findings of this study show that AnxA6 modulates Wnt/ß-catenin signaling in articular chondrocytes. Since low Wnt/ß-catenin signaling activity is required for articular cartilage homeostasis and maintenance [8], one can speculate that AnxA6 in articular chondrocytes may play a role in the regulation of Wnt/ß-catenin signaling to keep its activity at the low levels required for cartilage homeostasis and maintenance. Any changes of AnxA6 expression, however, may alter Wnt/ß-catenin signaling activity in articular chondrocytes and ultimately may affect articular chondrocyte function and phenotype.

Our findings show that IL-1ß treatment resulted in much higher expression levels of catabolic markers and MMP-13 in human artiular chondrocytes than Wnt3a treatment, whereas Wnt3a treatment was more effective than IL-1ß in inhibiting aggrecan and type II collagen expression. Combined IL-1ß and Wnt3a treatment of human articular chondrocytes resulted in lower expression levels of catabolic markers and MMP-13 than IL-1ß treatment, whereas the combined treatment resulted in the highest decrease of aggrecan and type II collagen expression. High expression of AnxA6 resulted in further increase in catabolic marker and MMP-13 expression in IL-1ß-treated or combined IL-1ß and Wnt3a-treated human articular chondrocytes, whereas high AnxA6 expression decreased catabolic marker expression and increased MMP-13, aggrecan and type II collagen expression in Wnt3a-treated human chondrocytes. These findings suggest that Wnt/ß-catenin and NF-κB signaling contribute to cartilage degradation during OA by affecting chondrocyte functions and phenotype in different ways. In addition, these two signaling pathways interact with each other and this interaction affects the activity of each pathway. Finally, our findings suggest that AnxA6 acts as an intracellular modulator of the activities of both signaling pathways and the cross-talk between these two signaling pathways.

Previous findings showing that both loss- and gain-of function of ß-catenin in articular cartilage of mice resulted in accelerated OA development and progression or chemical inhibitors of Wnt/ß-catenin signaling inhibited OA development and progression [6, 39–42], suggest that Wnt/ß-catenin signaling has to be tightly regulated in joint cells. Therefore, the findings of this study demonstrating that the lack of AnxA6 in articular chondrocytes stimulates Wnt/ß-catenin signaling, whereas overexpression of AnxA6 inhibits Wnt/ß-catenin signaling suggest that AnxA6 and its expression levels in articular chondrocytes may play a role in the tight regulation of Wnt/ß-catenin signaling activities. In addition, it is possible that in a disease, such as OA, numerous signaling pathways affect cartilage degradation and that the interactions or crosstalk between these various signaling pathways are major contributors to cartilage degradation. For example, it is possible that increased Wnt/ß-catenin signaling in AnxA6-/- mice may result in decreased NF-κB activity. This possibility is supported by our previous findings showing that loss of AnxA6 function resulted in a slower development and progression of post-traumatic OA after partial meniscectomy [20].

In summary, AnxA6 expression in human articular chondrocytes affects Wnt/ß-catenin and NF-κB signaling activities and the cross-talk between these two signaling pathways. Specifically, our findings reveal that AnxA6 expression in chondrocytes stimulates NF-κB signaling via direct interaction with p65 of the active NF-κB complex as shown previously by us [20], and indirectly via the inhibition of Wnt/ß-catenin signaling. Wnt/ß-catenin signaling has been shown in a previous study and the current study to inhibit NF-κB activity in human articular chondrocytes [17]. In addition, we show that specifically high expression of AnxA6 stimulates catabolic and inflammatory events in IL-1ß and IL-1ß/Wnt3a-treated articular chondrocytes, suggesting that AnxA6 may be involved in the regulation of catabolic and inflammatory events in articular chondrocytes by modulating the activities and the cross-talk of these two signaling pathways.
